# Bile Acids in Pancreatic Carcinogenesis

**DOI:** 10.3390/metabo14070348

**Published:** 2024-06-21

**Authors:** Bharti Sharma, Kate Twelker, Cecilia Nguyen, Scott Ellis, Navin D. Bhatia, Zachary Kuschner, Andrew Agriantonis, George Agriantonis, Monique Arnold, Jasmine Dave, Juan Mestre, Zahra Shafaee, Shalini Arora, Hima Ghanta, Jennifer Whittington

**Affiliations:** 1 Department of Surgery, NYC Health + Hospitals/Elmhurst, New York, NY 11373, USA; twelkerk1@nychhc.org (K.T.); nguyenc15@nychhc.org (C.N.); elliss5@nychhc.org (S.E.); bhatian1@nychhc.org (N.D.B.); kushnerz@nychhc.org (Z.K.); agriantg@nychhc.org (G.A.); davej@nychhc.org (J.D.); mestreju@nychhc.org (J.M.); shafaeez1@nychhc.org (Z.S.); arorash@nychhc.org (S.A.); ghantah1@nychhc.org (H.G.); harrisj20@nychhc.org (J.W.); 2 Icahn School of Medicine at Mount Sinai, New York, NY 10029, USA; agrianta@nychhc.org (A.A.); monique.arnold@mountsinai.org (M.A.)

**Keywords:** pancreatic cancer, bile acids, genetic alteration, tumorigenesis

## Abstract

Pancreatic cancer (PC) is a dangerous digestive tract tumor that is becoming increasingly common and fatal. The most common form of PC is pancreatic ductal adenocarcinoma (PDAC). Bile acids (BAs) are closely linked to the growth and progression of PC. They can change the intestinal flora, increasing intestinal permeability and allowing gut microbes to enter the bloodstream, leading to chronic inflammation. High dietary lipids can increase BA secretion into the duodenum and fecal BA levels. BAs can cause genetic mutations, mitochondrial dysfunction, abnormal activation of intracellular trypsin, cytoskeletal damage, activation of NF-κB, acute pancreatitis, cell injury, and cell necrosis. They can act on different types of pancreatic cells and receptors, altering Ca^2+^ and iron levels, and related signals. Elevated levels of Ca^2+^ and iron are associated with cell necrosis and ferroptosis. Bile reflux into the pancreatic ducts can speed up the kinetics of epithelial cells, promoting the development of pancreatic intraductal papillary carcinoma. BAs can cause the enormous secretion of Glucagon-like peptide-1 (GLP-1), leading to the proliferation of pancreatic β-cells. Using Glucagon-like peptide-1 receptor agonist (GLP-1RA) increases the risk of pancreatitis and PC. Therefore, our objective was to explore various studies and thoroughly examine the role of BAs in PC.

## 1. Pancreatic Cancer

The pancreas, a retroperitoneal organ, is composed of exocrine and endocrine cells [1]. Around 80% of the tissue mass forms the exocrine pancreas. It comprises acinar and duct cells. Centro acinar cells are located near the ducts in the acinar cells. The acinar cells are responsible for synthesizing and secreting zymogens into the ductal lumen [2]. They assist in the production of isotonic, alkaline pancreatic juice (pH 8), consisting of enzymes such as amylase and trypsin [3], which is essential for food digestion [4]. It plays a crucial role in regulating protein, carbohydrate digestion, and glucose homeostasis. On the other hand, the endocrine pancreas contributes to hormonal secretion, thus regulating glucose homeostasis and glandular secretions [5]. Islets of Langerhans have alpha, beta, delta, epsilon, and upsilon cells [6]. They are involved in several hormonal products like glucagon, somatostatin, proinsulin, insulin, amylin, pancreatic polypeptide (PP), and C-peptide, and perform endocrine functions [7].

**Figure 1:**
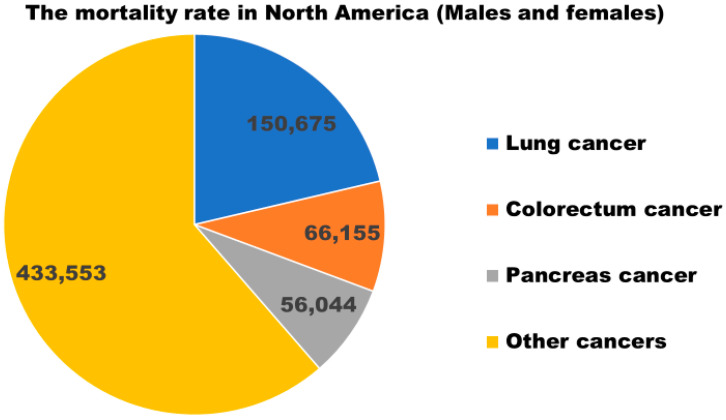
Number of mortality cases associated with pancreatic cancer in the year 2022 (values are taken from Globocan, and the graph was designed by the authors) [10].

Pancreatic cancer (PC) is an aggressive disease, accounting for 7% of deaths in cancer patients [8], and is the third leading cause of cancer-related death in males and females and is projected to become the second by 2030 [9]. In 2020, 466,003 (4.7%) cases were diagnosed with PC [10]. In 2022, the number of cases with PC-associated mortality within North America in both genders was found to be 56,044, as shown in Figure 1 [10]. An estimated number of 64,050 new cases and 50,550 deaths were associated with pancreatic cancer in 2023 [11]. Pancreatic ductal adenocarcinoma (PDAC) is the most common form of pancreatic cancer (PC), as it represents around 90% of the cases [12]. With no induced changes in hormonal secretion, it originates from exocrine tissue [12]. Primarily, PC does not give rise to obstructive symptoms or pain because of the large surrounding pancreatic space. This can be one of the explanations for why PC is diagnosed at late, inoperable, and incurable stages [1]. At present, there is no best way to screen PC at an earlier stage. Identification and systematic examination of individuals at risk of developing PC is the only diagnostic approach [13]. PC is a malignant digestive tract tumor considered to have the worst prognosis, with rising morbidity and mortality rates [14]. Identifying precursor lesions can help understand genomic characteristics associated with PC progression from earlier to advanced stages [15]. Non-invasive, pancreatic intraepithelial neoplasia (PanIN) lesions can be classified into PanIN-1A, PanIN-1B, and PanIN-2, whereas PanIN-3 is an advanced lesion. These all have diverse cytology and architecture. PanIN1-A is a flat lesion with low-grade dysplasia, PanIN1-B is a micropapillary type of lesion with low-grade dysplasia, and PanIN-2 has a frequent papillary formation with cell enlargement and nuclear crowding, and is hyperchromatic with lack in polarity. PanIN-3 exhibits luminal necrosis and severe nuclear atypia [16,17]. Low-grade lesions can be found in patients with chronic pancreatitis and observed with low risk of PC [18]. High-grade PanIN-3 lesions are observed with a high risk of PC and are found in patients with invasive PDAC [18]. Around 60% of PCs are prevalent in the pancreatic head, which is near various bile tracts [19]. Maintaining the idea that bile acids (BAs) play an important role in PC development [20,21], we performed a thorough literature search to bring forth this review describing the contribution of BAs in PC.

Several studies have reported that the development and progression of PC is associated with numerous risk factors. Some of these risk factors are obesity, alcohol consumption, radiation, dietary factors, race, gender, smoking, blood group, occupational hazards, age, genetic aberrations, family history/ hereditary pancreatitis, ethnicity, chronic pancreatitis [22], Peutz–Jeghers syndrome, gall stones [1], hormonal abnormality, allergy, and diabetes mellitus [12,23,24]. Risk factors like diet, smoking, and alcohol consumption can be controlled and are called modifiable, whereas, age, blood group, gender, genetic aberrations, and family history/ hereditary pancreatitis are a few examples of non-modifiable risk factors [25]. Acute pancreatitis (AP) is considered to be an early symptom of PC [26]. Based on studies, a survival rate of 20% in patients diagnosed with PC, compared to 28% in patients diagnosed with PC and AP (both), has been recorded over one year [27]. Dietary fat can induce BA secretion into the duodenum and elevate the fecal BA concentration [28]. Absorption of dietary fat, fat-soluble vitamins, and regulation of cholesterol metabolism can be affected by BAs [28]. Proper functioning of intestinal tight junctions and trans-epithelial permeability is regulated by normal intestinal flora by redistributing Toll-like receptor 2 protein (TLR-2) [29]. BAs can alter intestinal flora due to a high-fat diet leading to mucosal permeability [29]. High permeability leads BAs into blood circulation, allowing the translocation of gut microbes and associated products into the bloodstream, followed by chronic local and systemic inflammation [30]. High anti-oxidants in fruits and vegetables can help reduce inflammation and oxidative stress [31] caused by various PC-associated risk factors [32].

Tobacco smoking is one of the many important causes that favor PC development [33]. According to Talimini et al. (1999), the smoker population is presented with a severe risk of developing PC when compared with non-smokers [34]. Hermann et al. (2014) revealed the effects of nicotine on PC development in a mouse model with active forms of Kirsten rat sarcoma virus gene (KRAS) expression. In their study, nicotine-activated AKT-ERK-MYC signaling led to dedifferentiation, loss of differentiation in acinar cells, enhanced aggressiveness in cancer cells and increased numbers of circulating cancer cells, hyperactivation of oncogenic KRAS, and inhibition of Gata6 promoter activity accompanied by loss of GATA6 protein, altered gene expression and functional characteristics [35]. Protein kinase B (PKB), also known as Akt, is a group of three serine/threonine-specific protein kinases. It plays a crucial role in various cellular processes such as cell migration, regulation of gene expression, cell survival, and cell proliferation. Another important protein, extracellular signal-regulated kinase (ERK), belongs to the mitogen-activated protein kinase family and is involved in controlling blood vessel constriction and the growth of vascular smooth muscle cells. Additionally, the MYC proto-oncogene is a critical molecular factor in both the initiation and perpetuation of tumorigenesis.

**Figure 2:**
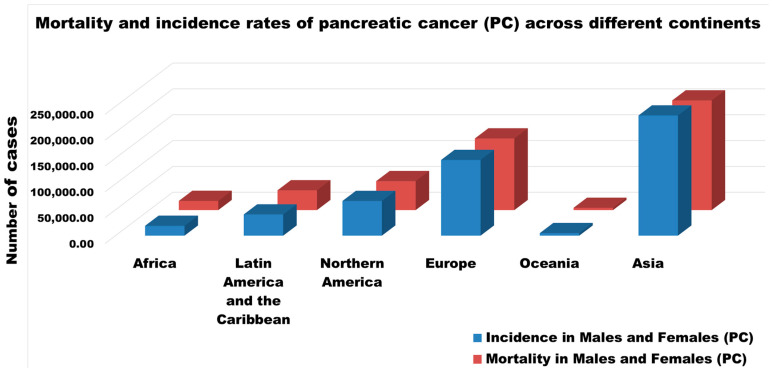
Number of cases showing mortality and incidence rates of pancreatic cancer (PC) across different continents in the year 2022 (values are taken from Globocan, and the graph was designed by authors) [10].

Some PC-associated mutations stimulated by nicotine include those of KRAS, p53, COX-2, p16INK4A (also known as P16 and MTS1), and SMAD4 [35,36]. Alcohol consumption can stimulate blood and intestinal BA levels [37] by two pathways [38]: first by increasing cholesterol 7α-hydroxylase synthesis [38,39], and second, by reducing feedback inhibition of BA synthesis by interrupting the enterohepatic circulation of BAs [37]. Different research models have been developed by researchers to explore various aspects of PC. For example, morphological and genetic observation, when combined, can serve as a progression model for PC [40]. Depending on the cancer history of an individual’s family, risk prediction statistical models such as PancPRO (a statistical model) can help understand the risk related to PC development [41,42]. Based on various heritability studies, >20% of PC cases are due to variations in inherited sequences [43,44,45,46]. According to the International Agency for Research on Cancer (IARC), in 2022, the highest PC-associated mortality and incidence rates in both genders were found in Asia, followed by Europe, North America, Latin America, the Caribbean, Africa, and Oceania, as shown in Figure 2 [10]. Among white and Asian populations, KRAS is the most frequently mutated gene, followed by TP53, whereas in Black or African American populations, TP53 is the most mutated gene, followed by KRAS [47]. In addition to KRAS and TP53, several other genes are mutated in PC. We have attempted to explore the interactions between these genes using various exploratory tools [47,48]. The development of PC can be affected by inflammation of the Islets of Langerhans, products of activated macrophages, neutrophil granulocytes, diabetes, reactive oxygen species (ROS), insulin resistance, and growth promotion [49].

## 2. Genetic Alterations in PC 

A frequently occurring form of PC is PDAC [50]. Generally, a nonmalignant fibrotic pancreatic tissue revealing atrophy and dilated ducts surrounds PDAC [51]. Some PDACs present as firm white-yellowish masses of the pancreatic head with poor demarcation [52]. Overall, the 5-year survival rate of PC patients is reported as very poor, i.e., ~11.5% [53]. KRAS, a proto-oncogene, is involved in the proliferation, differentiation, metabolism, and survival of cancer cells. From 90% to 95% of PC cases are seen with KRAS mutation [52]. Mutations in the KRAS gene can occur when a single nucleotide base is changed, inserted, or deleted in the DNA or RNA sequence of an organism. These mutations often happen at codon 12 (G12), codon 13 (G13), or codon 61 (Q61). The most common mutation, G12D, is present in 40% of pancreatic cancer patients. This mutation results in a GAT sequence replacing the normal GGT sequence, leading to the production of aspartic acid instead of glycine. Other prevalent mutations include G12V, which produces valine, and G12R, which produces arginine. The inactivation of tumor-suppressing genes such as SMAD4, P53, P16, and PTEN promotes the initiation and development of PC [54]. Some genes are frequently mutated, whereas some are rare. Mutations of a proto-oncogene (KRAS) and tumor suppressors (TP53, SMAD4) are frequent in PC and associated with cell cycle dysregulation [55]. Mutations of tumor suppressors like BRCA and mismatch-repairing genes, such as LKB1/STK11, AKT (AKT2), or Protein kinase B (PKB) (serine-threonine kinases), are rare genetic events [56].

**Figure 3:**
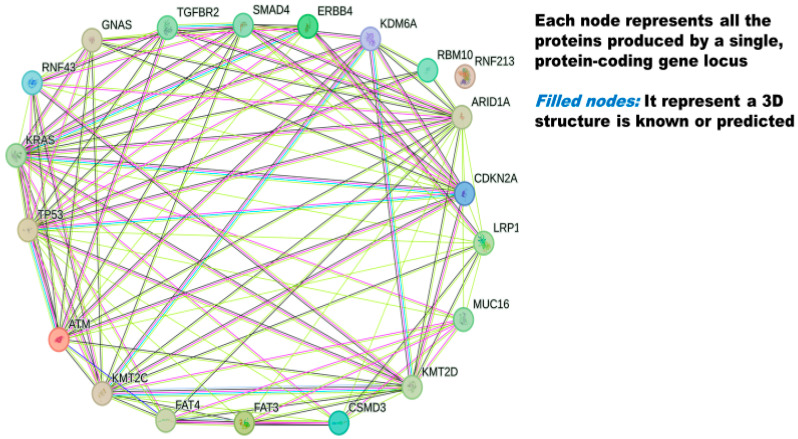
The 20 most frequently mutated genes in pancreatic cancer (PC). Names of genes are extracted from the NIH National Cancer Institute GDC data portal (explained in Table 1 of this paper) [47]. A string figure showing protein-to-protein association was created using STRING [48].

Based on genomic analysis, KRAS, CHEK2, BARD1, BRCA1, and BRCA2 [57], the DNA mismatch repair (MMR) genes MLH1, MSH2, MSH6, and PMS2 [58], and CDKN2A, NBN, SMAD4, ATM [59], PALB2 [60] STK11 [61], TP53, and MUTYH/MYH [62] are some of the genes associated with PC. Among these, TP53, KRAS, SMAD4, and CDKN2A are four major driver genes of PC [63,64,65]. SWI/SNF complexes are PDAC epigenetic drivers with multi-subunit complexes [66]. They are involved in chromatin remodeling, DNA repair, and regulation of transcription [66]. Genes like ARID1B, ARID2, PBRM1, SMARCA2, and SMARCA4 (also called transcriptional activator BRG1) are associated with the encoding of multi-component SWI/SNF complexes. As reported by various studies, these encoding genes are mutated in human PDAC [63,67,68,69,70]. Interactions between some of the commonly occurring mutated genes are shown in Figure 3, and functions of these genes in a non-mutated (healthy) form are shown in Table 1 of this paper.

Dysregulation in signaling pathways, oncogenes, and tumor suppressor genes contributes to the malignancy of PC [23]. In PC, high incidences of RAS mutations are identified [71]. In a study conducted by Jones et al. (2008), ~63 genetic alterations were found in PC. These alterations were associated with 12 pathways and processes such as DNA damage control, wingless-type MMTV integration site family (Wnt), neurogenic locus notch homolog protein (Notch), apoptosis, KRAS, small GTPase signaling, integrin, hedgehog, invasion, homophilic cell adhesion, Jun N-terminal kinase (JNK), control of G1/S phase transition, and transforming growth factor-β (TGF-β) [64]. KRAS and Wnt have an important role in cell proliferation and transcription, TP53 contributes to apoptosis, and SMAD, P16, and CDKN2A are regulators of the cell cycle [23]. Mutation of KRAS, neuroblastoma RAS viral (v-ras) oncogene homolog (NRAS), and Harvey rat sarcoma viral oncogene homolog (HRAS) are located in codon 12, with a frequency of 20 to 100% in tumor progression [72,73]. Polyphenic effects including cell proliferation, migration, and survival are promoted by small GTP-binding cytoplasmic proteins encoded by RAS family proteins [74]. Based on studies, KRAS-mutated PC cell lines such as MiaPaca and Capan1 are often identified with loss of the wildtype KRAS allele [72]. In cancer cell lines like Panc1 and SU8686, mutated alleles, when compared with wild-type alleles, are presented with suppressed expression [72]. With the loss of the wildtype allele and late occurrence, a missense mutation in sequence coding of TP53 is reported in over 50% of cases diagnosed with PC [75,76,77,78,79,80]. Heavily glycosylated proteins known as mucins (MUC) possess the ability to build selective molecular barriers at the epithelial surface and are crucial for regulating morphogenesis. These proteins contribute to cellular growth, adhesion, differentiation, immunity, transformation, and invasion [81,82,83]. Twenty-one mucin genes are found in humans and used as potential diagnostic tools for PC. In PC, MUC4, 5AC, and 1 are revealed to be highly expressed and associated with poor outcomes. These genes can serve as promising biomarkers for PC progression [84,85,86,87]. BAs can induce changes in the expression of mucins and play an important role in cancer progression [87,88,89,90,91,92]. As reported by studies, MUC4 undergoes overexpression in the presence of BAs and enhances the carcinogenic potential of PDAC cells [93].

BAs can act on different types of pancreatic cells, for example, duct cells, and can alter ductal secretion by inducing pathological Ca^2+^ signals [94,95,96]. A large Ca^2+^ signal aberration in pancreatic acinar cells is found to be caused by BAs like taurolithocholic acid 3-sulfate (TLC-S). This allows depletion of intracellular Ca^2+^ stores as well as enhanced entry of Ca^2+^ [97]. In acinar cells, an increase in Ca^2+^ is associated with cell necrosis and vacuolization, as well as untimely intracellular enzyme activation [98]. BAs can act as pathological agents, and their signaling can affect various pathological conditions [99]. They can give rise to mitochondrial dysfunction, abnormal activation of intra-cellular trypsin, cytoskeletal damage, activation of nuclear factor- kappa B, acute pancreatitis, cell injury, and cell necrosis [100,101,102,103,104].

The pancreas has both exocrine and endocrine functions [105]. Acute pancreatitis (AP) is associated with the development of PC [106]. Pancreatic stellate cells (PSCs) not only interact with cancer cells but are also related to pancreatic fibrosis [107]. According to Xu et al. (2010), PSCs can promote cancer metastasis [108]. The stromal reaction produced by PSCs can enhance the development and progression of PC [109,110,111]. As reported by Pries et al. (1983), taurocholate is a suppressor of BA production and is more potent than cholate [112]. In a study by Ferdek et al. (2016), cholate and taurocholate were shown to be inducers of necrosis and Ca^2+^ signaling in stellate cells. Acinar cells are reported to be affected by taurolithocholic acid 3-sulfate. Extracellular Ca^2+^ is one of the core requirements to mediate Ca^2+^ signals and necrosis [113]. Bradykinin-induced signals in stellate cells can promote pancreatic damage mediated by BAs and have crucial involvement in acute biliary pancreatitis [113]. Platelet endothelial cell adhesion molecule-1 (PECAM-1), also known as cluster of differentiation (CD31), is important for cellular immunity, cell proliferation, migration, and apoptosis [114]. In a current study, staining of endothelial cell marker CD31 revealed an increase in endothelial cell number and confirmed the presence of CD31 in the peritumoral stroma of PC [115].

## 3. What Are Bile Acids?

Bile is a yellow-greenish fluid, synthesized in liver hepatocytes, carried to the duodenum via bile ducts, and helps in lipid metabolism [116]. Its constituents are bile acids [117], cholesterol, amino acids, vitamins, lecithin, toxins, bilirubin, and heavy metals [118]. BAs conjugated with glycine or taurine are involved in the synthesis of bile salts. Thus, BAs are building blocks of bile salts. These are saturated, hydroxylated C-24 cyclopentanophenanthrene sterols [119]. These are synthesized from cholesterol in perivenous hepatocytes surrounding the central hepatic vein [120], and affiliated with cholesterol-derived sterols [121]. They are crucial for dietary lipid solubilization and absorption of fat-soluble vitamins such as A, D, E, and K [122]. BAs are natural products that can be isolated in pure form [123]. Hydroxylation of the steroid ring and the presence of the carboxyl group side chain make BA polarity higher than that of cholesterol [121]. Due to the amphipathic character of BAs, they are known as natural detergents [121]. They are strong digestive surfactants that act as emulsifiers to promote lipid absorption [124].

The main constituents of BAs are organic molecules (phospholipids, proteins, bile salts, cholesterol), water, and electrolytes [125]. The release of stored bile from the gallbladder depends on bile flow in the duodenum [116]. The hormone Cholecystokinin (CCK) regulates bile flow in the duodenum [116]. Any type of blockage in the extrahepatic biliary system can result in biliary obstruction [126]. Biliary obstruction is one of the core characteristics of PC [116]. It can result not only in renal failure and hepatic dysfunction but also in infections, bleeding complications, and nutritional inadequacy [126]. BAs are formed as an end-product of cholesterol catabolism [127,128]. They act as nutrient signaling hormones by activating receptors such as nuclear receptors (pregnane X receptor, farnesoid X receptor) and G-protein coupled receptors (muscarinic receptors, sphingosine-1 phosphate receptor 2) to promote digestion, transportation, and metabolism of various nutrients [129]. BAs are important for the absorption and excretion of cholesterol as well as the maintenance of plasma cholesterol levels [130]. Farnesoid X receptor (FXR) serves as a critical nuclear receptor activated by bile acids (BAR) and is predominantly expressed in the liver and intestine. When FXR becomes active in the liver, it triggers the enhanced expression of specific target genes. These genes encompass ATP-binding cassette, sub-family B member 11 (ABCB11), which plays a key role in the bile salt export pump, and ATP-binding cassette, sub-family B member 4 (ABCB4), which serves as a phospholipid transporter. These transporters function to decrease the levels of bile salts and lipids within cells by accelerating their transport into the bile. FXR activation depends on the activation of the AKT signaling pathway [131]. BAs act on the FXR target gene known as small heterodimeric partners (SHP) [131] and possess the ability to deorphanize BAs associated with FXR [132]. Activation of Protein kinase C, zeta (PKCζ) is reported as a helping act of taurocholic acid (TCA) towards activation of SHP [132]. As discussed above, the synthesis of BAs takes place in the liver. Their metabolism involves hepatic endogenous and xenobiotic metabolism along with interaction with constitutive androstane receptor (CAR) and PXR [133]. Their excretion involves multiple organs but at present, physiological regulation of BA metabolism is unknown [134]. BAs can disrupt the mucosal barrier to diffusion [135] and are considered as pro-carcinogenic molecules [136,137,138].

Bile is constituted by greater than 60% of glycine-conjugated BAs (pKa values of 4.3–5.2) and ~20% of taurine-conjugated BAs (pKa values of 1.8–1.9) [117]. The ratio between both BAs is 3:1 [139]. Taurine-conjugated BAs can act as carcinogens [139]; their properties such as solubility, frequent cell contact, and cross-talk upgrade their carcinogenicity [139]. Non-conjugated BAs are more carcinogenic than conjugated BAs [139]. Approximately 95% of BAs undergo intestinal (terminal ileal) active reabsorption and are carried to the liver [140]. The level of BAs in plasma can be co-related with a fecal concentration of BAs [141]. An enhanced level of BAs such as hydrophobic 12a-hydroxylated BAs and deoxycholic acid in type 2 diabetes have been reported in the literature [142]. BAs act as ligands for various receptors like FXR [143], PXR, vitamin D receptor, and androstane receptor [144]. For example: chenodeoxycholic acid (CDCA) acts as a potent agonist for FRX, whereas deoxycholic acids (DCA) and lithocholic acid (LCA) act with low affinity on the same receptor [143]. Expression of FXR is majorly reported in reproductive tissues, liver, kidney, pancreas, reproductive tissues, and intestines [145].

Synthesis of BAs is a multistage process [146] that includes a series of enzymatic reactions [147]. Primary pathways for cholesterol catabolism are represented by BAs [148]. In BA-associated synthetic pathways, immediate products are termed primary BAs such as chenodeoxycholic acid and cholic acid. Intestinal bacterial flora act on these primary BAs to form secondary BAs such as lithocholic acid and deoxycholic acids [147]. Formation of primary BAs involves cholesterol 7α-hydroxylase (CYP7A1), a cytochrome P450 enzyme that promotes hydroxylation of cholesterol [147,148,149,150]. CYP7A1 controls the conversion of cholesterol to BAs. Another pathway of BA formation is an “acidic or alternative” pathway controlled by CYP27A1. It mediates the conversion of oxysterols to BAs [147,148]. CYP7A1 is regulated by BAs, whereas CYP27A1 is not. The conversion of BA intermediates into chenodeoxycholic acid or cholic acid is controlled by CYP8B1 [147]. The overall hydrophobicity of the BA pool is determined by the ratio of cholic acid to chenodeoxycholic acid. CYP8B1-mediated hydroxylation assists in the formation of hydrophilic cholic acid molecules [147]. There are 17 sets of enzymes in hepatocytes that are essential for the removal of side chains, steroid core modification, and formation of a conjugated form of taurine or glycine [151]. Passive or carrier-mediated transport processes mediate the reabsorption of BAs into the intestinal proximal region [152,153], whereas apical sodium-dependent bile acid transporter (ASBT) supports the recovery of BAs in the distal ileum [154].

## 4. Contribution of Bile Acids in Pancreatic Cancer

In 1940, BAs (deoxycholic acid, deoxycholate) were reported to induce cancer in rodents and were proposed as carcinogens [155]. Rodents, when induced with BAs, presented with malignant spindle-celled tumors. Epidemiological studies confirmed an association between BAs and cancer [156]. Both primary and secondary BAs are contributors to tumorigenesis, and the level of variations strictly depends on the cancer type [157].

The role of BAs in PC is not clear [158]. An increase in BA level can elevate ROS production, oxidative stress, cell membrane damage, activation of downstream signaling (EGFR, NF-κB, PKC), and DNA mutations. This promotes aggressive neoplastic cell growth in organs such as the stomach, colon, and others [157]. Enhanced levels of BAs can result in BA reflux in the pancreatic duct and can affect acinar cells, thus promoting pancreatic adenocarcinoma progression [158]. Factors such as smoking, alcohol consumption, and high-fat diet possess the ability to elevate BA levels. BA-associated dysregulated metabolism can result in gallstone formation [158].

Secretion of BAs is strongly regulated by gastric acid. As reported by Adachi et al. (2006), bile reflux into the pancreatic ducts can lead to accelerated kinetics of epithelial cells and promote the development of pancreatic intraductal papillary carcinoma (IPC) [159]. Based on studies, BAs can induce pancreatic adenocarcinoma and mediate progression at multiple stages [160]. Pancreatitis caused by restricted bile flow (which occurs in gallstone formation) is a risk factor for pancreatic adenocarcinoma [158,161,162]. Pre-malignant pancreatic ductal cells, on treatment with BAs, can result in tumorigenesis [159,163].

BAs can increase the expression of COX-2 or mucins and can mediate the development of cancer [84,163,164]. Higher levels of BAs such as unconjugated cholic acids were reported by Rees et al. (2017) in patients with adenocarcinoma of the pancreas. Such studies promote an understanding of cancer biology and the role of metabolites such as BAs in cancer cells [165]. In a study conducted by Sarkar et al. (2023), levels of sphingosine-1-phosphate receptor 2 (S1PR2) and PC progression were shown to be raised by conjugated bile acids (CBAs) [166]. BAs in PC are associated with dysregulation of the cell cycle, cell membrane disruption, activation and expression of inflammatory mediators, and reduction of apoptosis [21,158]. In multiple studies, the occurrence of PDAC has been reported majorly in pancreatic heads from ductal cells. With tumor progression, the flow of bile is hindered, leading to the development of obstructive jaundice. It elevates the serum level of BAs. Enhanced levels of BAs have carcinogenic potential and can result in gastrointestinal cancer [167].

**Figure 4:**
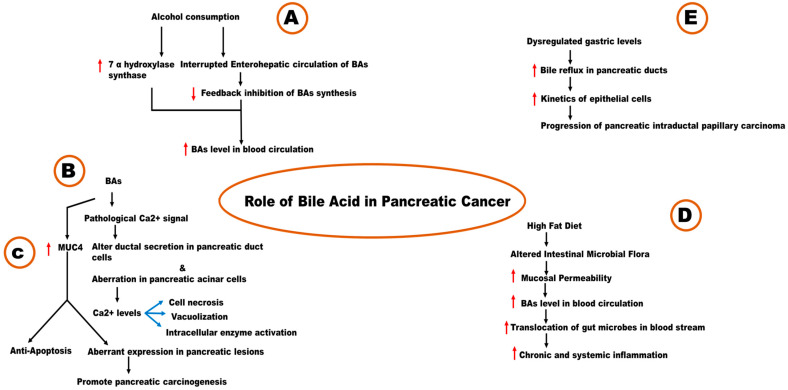
BA mechanisms of action in the progression of pancreatic cancer (Arrows facing upwards (in red) mean increase and arrows facing downwards (in red) mean decrease).

In a study conducted by Gál et al. (2020), BAs were able to induce MUC4 overexpression and promote carcinogenesis [93]. They reported an elevated level of serum BAs such as taurochenodeoxycholic acid, glycochenodeoxycholic acid, glycocholic acid, and taurocholic acid in patients diagnosed with PDAC [93]. Overexpression of MUC20 and 1 are associated with poor survival in PDAC patients [168,169]. The presence of MUC5B, 13, and 5AC can be found in PDAC and pancreatic intraepithelial neoplasia, whereas they are absent in normal pancreas [170]. Aberrant expression of MUC17 in PC is not uncommon [171,172]. MUC4 is reported with aberrant expression in premalignant and malignant pancreatic lesions [173,174,175,176]. It can act as an intramembrane ligand for v-erb-b2 avian erythroblastic leukemia viral oncogene homolog 2 (ERBB2/Erb-B2/HER2/neu) and promote antiapoptotic function of MUC4 [177]. It can alter the actin organization, enhance invasiveness, and inhibit integrin-mediated cell adhesion. Silencing of MUC4 can dysregulate genes associated with growth and metastasis: for example, plakoglobin, caspase 3, 2, and 7, thrombomodulin, neuregulin-2, Liver Intestine-cadherin (LI-cadherin), S100 calcium-binding protein A4 (S100A4), AnnexinA1 (ANXA1), Ras-related C3 botulinum toxin substrate 1 (RAC1), and carcinoembryonic antigen cell adhesion molecule 6 (CEACAM6) [85]. The contribution of BAs in PC is explained in Figure 4 of this paper.

Iron is one of the many crucial trace elements in the body. BAs possess the ability to solubilize iron in the duodenum and promote its absorption [123]. Bile salts have cholanic ring 7 alpha-OH and/or 12 alpha-OH groups that afford high affinity towards iron [178]. In 2012, Dixon et al. first proposed iron-mediated cell death known as ferroptosis (FPT). It is a new mode of non-apoptotic cell death [179]. In FPT, ROS such as peroxides (ROOH and H_2_O_2_), superoxide (O_2_•), and free radicals (RO• and HO•) [180] are generated enormously by Fenton reaction (Fe^2+^ reacts with hydrogen peroxide) [181]. Based on various studies, FPT has been identified in PC [182]. Higher levels of iron may result in lipid peroxidation (LP) [183]. LP is one of the characteristics of FPT [179]. Hence, connecting these dots, there is a possibility that BAs are associated with FPT in PC and contribute to its growth.

Gastrointestinal microbial flora contains around 1014 bacteria and is associated with 99% of multi-functional genes [184]. Microbial flora is associated with the size and composition of the BA pool, and the BA pool can affect the diversity of gut microbiota [185]. The gut microbiome comprises various phyla and genera. Examples of phyla include *Bacteroidetes*, *Firmicutes*, *Fusobacteria*, *Proteobacteria*, *Verrucomicrobia*, and *Actinobacteria*. Examples of genera are *Clostridium*, *Pepto streptococcus*, *Bacteroides*, *Lactobacillus*, *Bifidobacterium*, *Methanobrevibacter*, *Ruminococcus*, *Eubacterium*, and *Propionibacterium* [186]. Based on a study conducted by Nejman et al. (2020), bacterial DNA is identified in more than 60% of PC. The sources of this DNA include *Klebsiella pneumoniae*, *Fusobacterium nucleatum*, *Enterobacter asburiae*, and *Citrobacter freundii* [187]. Poly-β-1,6-N-acetyl-d-glucosamine (PNAG) is a bacterial surface polysaccharide and an essential component of biofilm [188]. In *K. pneumoniae*, the formation of biofilm and production of PNAG are stimulated by bile salts [189].

The BA pool and microbial flora have been shown to work closely along with higher chances of contributing to PC growth and development. A total BA pool of around 1.5–4 g undergoes recycling 4–14 times every day with a recovery rate of 95% in enterohepatic circulation and a contribution of 5% to fecal loss [190]. Microbiota plays an important role in the transformation of primary BA to secondary BA [185]. Functional-centered changes in gut microbiota can negatively influence BA levels and are associated with the development of cancers such as PC [157,186]. For example, infection by *C. freundii* can cause disbalance in intestinal microbiota, bile acid synthesis, and pathogenic bacterial colonization. This results in inflammation and the disruption of tissue structure [191].

Based on various studies, Glucagon-like peptide-1 (GLP-1) is vigorously secreted by BAs [192]. GLP-1 is a long peptide hormone comprising 30–31 amino acids. Its effect is mediated by various GLP-1 receptors located in the pancreas [193]. Studies have confirmed the involvement of GLP-1 in the proliferation of pancreatic β-cells [194,195,196]. GLP-1 mimetics such as exenatide and liraglutide have been reported with pancreatitis as one of their side effects [197,198,199,200]. The glucagon-like peptide-1 receptor (GLP-1R) is a G-protein-coupled receptor that is expressed particularly in pancreatic islet cells [201] bound to the plasma membrane of pancreatic acinar cells [202]. These are involved in the initiation and progression of cancer as well as associated oxidative stress and inflammation [193]. Glucagon-like peptide-1 receptor agonists (GLP-1RAs) work by pancreatic GLP-1 receptor activation [203]. Activation of GLP-1R directly contributes to cell proliferation and increases cell survival [204]. GLP-1RAs are associated with the regulation of crucial molecular pathways [193]. These are also involved in indirect cancer growth [205,206]. An endocrine neoplasm of the pancreas is called insulinoma [207]. Studies have revealed an immense expression of GLP-1R with an incidence rate of >90% on benign insulinoma cell surfaces [208,209]. A high risk of pancreatitis and PC is associated with GLP-1RAs [210]. In this aspect, BAs and GLP-1 are associated with PC growth and development. However, there is a need for detailed research aimed at shedding light on the contribution of BAs to the development of PC due to their interaction and involvement with GLP1.

## 5. Conclusions and Future Direction

Pancreatic cancer is often diagnosed at an advanced stage, making early detection uncommon. There is ongoing debate about whether elevated bile acids are harmful or beneficial for pancreatic cancer. However, bile acids are closely linked to the development of pancreatic cancer. They are associated with many risk factors for pancreatic cancer, including alcohol consumption, smoking, high-fat diet/obesity, gallstones, pancreatitis, diabetes, and hypertriglyceridemia. Aside from their systemic effects, bile acids also have local tissue effects and can directly activate cancer signaling pathways. In the future, bile acids are likely to be recognized as signaling molecules in pancreatic cancer. Understanding how bile acids promote the progression of pancreatic cancer can aid in the development of new therapeutic targets and effective strategies for diagnosis and treatment.

Multiple studies have suggested that bile acids may act as cancer promoters in pancreatic cancer. For instance, pancreatic ductal adenocarcinoma (PDAC) is often associated with elevated levels of bile acids in the bloodstream. However, the impact of bile acids on the progression of pancreatic cancer has not been comprehensively assessed. Many questions remain unanswered, and further research, including oncological and physiological experiments, is necessary to confirm the role of bile acids in the development of pancreatic cancer. Detailed research studies are increasingly important to improve our understanding of pancreatic cancer’s biology, with the role of metabolites such as bile acids being crucial.

Bile acids have the potential to induce changes in various cellular proteins, receptors, signaling pathways, and molecules. Moreover, they can affect normal calcium and iron levels in the body. However, research on the role of bile acids in iron metabolism or ferritin processing is limited and requires scientific investigation. Additionally, the relationship between Glucagon-like peptide-1 and its receptors must be explored to understand its contribution to pancreatic cancer. The connection between bile acids, microbiomes, and pancreatic cancer is an area that requires in-depth research. The specific connections between pancreatic cancer and bile acids in cancer cell biology have not been fully explored. Therefore, both laboratory research and clinical studies in this area are important. The results of clinical trials can complement and validate laboratory findings, ultimately benefiting patients with pancreatic cancer.

## Figures and Tables

**Table 1:** The 20 most frequently mutated genes in pancreatic cancer (PC). The gene names, full forms, and functions are extracted from the NIH National Cancer Institute GDC data portal [47].

Most Frequently Mutated Genes in PC (Top to Bottom)	The Function of Non-Mutated (Healthy) Gene
KRAS (GTPase Kras)	Ras proteins bind GDP/GTP and possess intrinsic GTPase activity. Plays an important role in the regulation of cell proliferation.
TP53 (Cellular tumor antigen p53)	Acts as a tumor suppressor in many tumor types; involved in cell cycle regulation; induces growth arrest or apoptosis depending on the physiological circumstances and cell type.
CDKN2A (Cyclin-dependent kinase inhibitor 2A)	Acts as a negative regulator of the proliferation of normal cells by interacting strongly with CDK4 and CDK6
SMAD4 (Mothers against decapentaplegic homolog 4)	Plays a central role in the balance between atrophy and hypertrophy.
MUC16 (Mucin-16)	Provides a protective, lubricating barrier against particles and infectious agents at mucosal surfaces.
RNF43 (E3 ubiquitin-protein ligase RNF43)	Acts as a negative regulator of the Wnt signaling pathway by mediating the ubiquitination, endocytosis, and subsequent degradation of Wnt receptor complex components Frizzled.
KMT2D (Histone-lysine N-methyltransferase 2D)	Methylates ‘Lys-4’ of histone H3 (H3K4me). H3K4me represents a specific tag for epigenetic transcriptional activation. Acts as a coactivator for estrogen receptor by being recruited by ESR1, thereby activating transcription
ARID1A (AT-rich interactive domain-containing protein 1A)	Involved in transcriptional activation and repression of select genes by chromatin remodeling (alteration of DNA-nucleosome topology)
CSMD3 (CUB and sushi domain-containing protein 3)	Involved in dendrite development
TGFBR2 (TGF-beta receptor type-2)	Transduces the TGFB1, TGFB2, and TGFB3 signals from the cell surface to the cytoplasm and regulates cell cycle arrest in epithelial and hematopoietic cells, control of mesenchymal cell proliferation and differentiation, wound healing, extracellular matrix production, immunosuppression, carcinogenesis
FAT3 (Protocadherin Fat 3)	May play a role in the interactions between neurites derived from specific subsets of neurons during development
LRP1B (Low-density lipoprotein receptor-related protein 1B)	Potential cell surface proteins that bind and internalize ligands in the process of receptor-mediated endocytosis
KMT2C (Histone-lysine N-methyltransferase 2C)	Histone methyltransferase that methylates ‘Lys-4’ of histone H3. H3 ‘Lys-4’ methylation represents a specific tag for epigenetic transcriptional activation. A central component of the MLL2/3 complex, a coactivator complex of nuclear receptors, is involved in transcriptional coactivation.
RNF213 (E3 ubiquitin-protein ligase RNF213)	Involved in angiogenesis
ERBB4 (Receptor tyrosine-protein kinase erbB-4)	Plays an essential role as a cell surface receptor for neuregulin and EGF family members and regulates the development of the heart, the central nervous system, and the mammary gland, gene transcription, cell proliferation, differentiation, migration, and apoptosis
FAT4 (Protocadherin Fat 4)	Plays a role in the maintenance of planar cell polarity as well as in the inhibition of YAP1-mediated neuro progenitor cell proliferation and differentiation
ATM (Serine-protein kinase ATM)	Activates checkpoint signaling upon double strand breaks (DSBs), apoptosis, and genotoxic stresses such as ionizing ultraviolet A light (UVA), acting as a DNA damage sensor.
RBM10 (RNA-binding protein 10)	May be involved in post-transcriptional processing, most probably in mRNA splicing
GNAS (Guanine nucleotide-binding protein G(s) subunit alpha isoforms Xlas)	Guanine nucleotide-binding proteins (G proteins) function as transducers in numerous signaling pathways controlled by G protein-coupled receptors (GPCRs)
KDM6A (Lysine-specific demethylase 6A)	Histone demethylase specifically demethylates ‘Lys-27’ of histone H3, thereby playing a central role in histone code

## Data Availability

Not applicable.
